# Utilization of a TiO_2_–CuO Bimetallic/Polyaniline Nanocomposite as a Transducer in a Solid Contact Potentiometric Sensor for the Determination of Vildagliptin

**DOI:** 10.3390/polym15193991

**Published:** 2023-10-04

**Authors:** Nehad A. Abdallah, Sameh A. Ahmed, Mohammed Almaghrabi, Yaser M. Alahmadi

**Affiliations:** 1 Pharmacognosy and Pharmaceutical Chemistry Department, College of Pharmacy, Taibah University, Al-Madinah Al-Mounawarah 30001, Saudi Arabia; shassan@taibahu.edu.sa (S.A.A.); mhmaghrabi@taibahu.edu.sa (M.A.); 2 Clinical and Hospital Pharmacy Department, College of Pharmacy, Taibah University, Al-Madinah Al-Mounawarah 30001, Saudi Arabia; yahmadi@taibahu.edu.sa

**Keywords:** polyaniline, transducer, metal oxides nanoparticles, potentiometry, vildagliptin, solid contact electrode

## Abstract

Current fundamental electrochemical research shows the potential of utilizing polymeric nanostructured materials as ion-to-electron transducers. In this paper, aniline was polymerized in the presence of TiO_2_ and CuO nanoparticles to yield a bimetallic/PANI nanocomposite. It was applied as a transducer in a carbon paste electrode for the potentiometric determination of vildagliptin in the presence of 18-crown-6-ether as a recognition element. The electrode’s potentiometric performance was studied according to the IUPAC guidelines. It exhibited a wide linearity range of 1 × 10^−2^ M to 1 × 10^−8^ M, remarkable sensitivity (LOD of 4.5 × 10^−9^ M), and a fast response time of 10 s ± 1.3. The sensor did not show any potential drift due to the absence of the water layer between the carbon paste and the metallic conductor. This endowed the sensor with high stability and a long lifetime, as 137 days passed without the need to change the carbon paste surface. The electrode was utilized for the determination of the concentration of vildagliptin in bulk, pharmaceutical tablets, and human plasma, with average recovery ranging from 97.65% to 100.03%.

## 1. Introduction

In recent years, the achievement of ultimate sensitivity and selectivity has become the main goal in the electrochemical analysis field. In addition, the miniaturization of potentiometric sensors has paved the way for the development of ion selective electrodes (ISEs) with smaller portable sizes, lower sample volumes, and less waste [1]. Solid contact ISEs (SC-ISEs) are considered the most attractive choice for developing a new sensor wherein the membrane is to be sandwiched between the solid contact transducer layer and the sample solution, which improves the mechanical flexibility and robustness of the electrode [2]. The polyvinyl chloride (PVC)-based membranes in SC-ISEs may suffer from limited stability and sensitivity due to the leaching of either the ionophore, ion-exchanger, or plasticizer into the sample solution, in addition to the possibility of the generation of a water layer near the transducing material leading to drift in the electrode potential. Therefore, PVC-membrane based sensors suffer from deteriorations in the potentiometric response, limitations in detection sensitivity, and short lifespans [1,3,4].

Carbon paste electrodes (CPEs) are solid contact electrodes that exhibit stable responses with low ohmic resistance. They are characterized by their ease of preparation and their high stability due to the solid nature of the electrode and lack of the internal filling solution. The ease of surface renewability of the carbon paste extends the lifespan of the electrode by three to four times more than the PVC-based ones. The carbon paste is composed of a graphite powder, analyte recognition element, ion-to-electron transducer, and a binder. The binder promotes the mobility and binding of the analytical species in sample solutions to the active surface of the electrode [5,6].

Improvements in the CPE response, stability, sensitivity, and intrinsic conductivity can be achieved through the modification of the transducer and the ion-recognition elements. Conducting polymers (CPs) have been widely used in the electrochemical field due to the π-electron configuration within the polymeric structure. They are characterized by their ease of synthesis, facilitation of room temperature operation, short response time, and high sensitivity, which make CPs promising materials in the electrochemistry field [2,7].

Polyaniline (PANI) has been a promising conducting polymer over the past few years due to its environmental stability, low cost, ease of synthesis, high sensitivity, and high electrical conductivity on doping with protonic acids. The conjugated electron system existing in PANI increases its conductivity in a favourable way so that it can be utilized as a battery electrode material, a transparent conductor, and, in biosensors, in corrosion protection practices [8]. To induce an ordered structure in the PANI polymer and improve its chemical stability and mechanical strength, other materials acting as fillers for the composite are required. Because of its unique features and applications in many electrical and electronic devices, the fabrication of PANI composites with various materials has gained a lot of interest [9]. In light of this, inorganic metal oxide nanoparticles are favourable candidates due to their intrinsic electronic properties, in addition to being used in the field of electrical applications [10]. The doping of nano-sized inorganic semiconductors into CPs yields many advantages, such as low thermal conductivity, high electrical conductivity, low energy consumption, a low detection limit, cost effectiveness, simplicity in fabrication, mass production, and extensive area processing. Mutual interactions between inorganic semiconductors and conducting polymers may result in fascinating features that differ greatly from those of the separate components [7,8]. Titanium oxide (TiO_2_) is an inorganic metal oxide that has specific characteristics, including high mechanical strength, high binding energy, high conductivity, good chemical stability, a large surface area, and nontoxicity, all of which favour its applicability in sensing devices [11,12,13,14,15]. Among all metal oxides, copper oxide (CuO) has attracted technological importance and has potential applications in electronic and optical applications [16,17,18]. Recently, a combination of PANI and single metal oxide nanoparticles were explored for use as ion-to-electron transducers. The metal oxide nanoparticles served as fillers to modify the PANI and improve its electrical and sensing properties compared to the plain PANI [7,19,20,21].

In a trial aiming to achieve an outstanding performance characteristics, a PANI/bimetallic nanocomposite was utilized as an ion-to-electron transducer in a potentiometric CPE where TiO_2_ and CuO nanoparticles were encapsulated in the shell of PANI, giving rise to a nanocomposite with a large surface area, high chemical stability, and sufficient mechanical strength. In this work, four CPEs were fabricated via the utilization of PANI, TiO_2_/PANI, CuO/PANI, and TiO_2_-CuO/PANI separately as a transducers in the presence of 18-crown-6-ether as a recognition element, and their potentiometric responses were monitored. The bimetallic nanomaterials that were combined with PANI exhibited better physical, chemical, and catalytic activity in comparison to the single metal oxide nanomaterials, meaning that they are promising transducers for the fabrication of robust and durable CPEs. Crown ethers were selected to act as host molecules and selectively bind to the guest analyte through the formation of host–guest inclusion complexes via dipole–dipole interactions [22].

**Figure 1:**
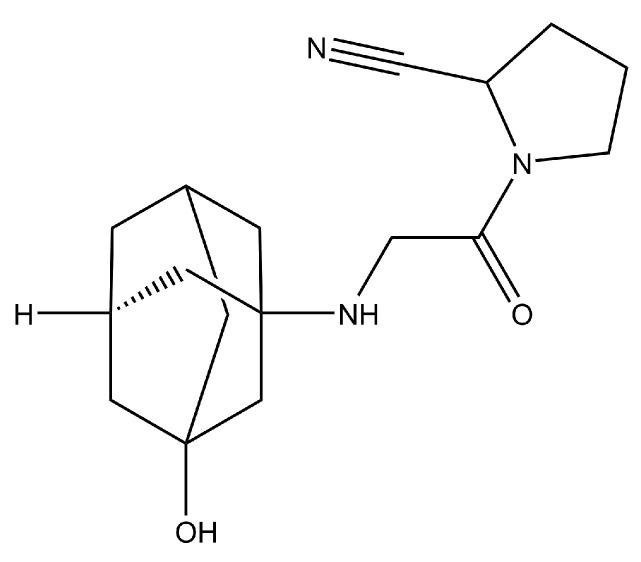
Chemical structure of vildagliptin.

Vildagliptin (Figure 1) is chemically named (S)-1-[N-(3-hydroxy-1-adamantyl) glycyl] pyrrolidine-2-carbonitrile, and it acts as a dipeptidylpeptidase-4 inhibitor (DPP-4). It is used orally for the treatment of type-II diabetes mellitus. DPP-4 inhibits glucagon-like peptide-1 (GLP-1) and glucose-dependent insulin tropic peptide (GIP) hormones. These hormones are vital for maintaining normal blood glucose levels by increasing glucagon and insulin release in the pancreas. Several analytical techniques have been reported in the literature for the determination of vildagliptin, such as spectrophotometry [23,24,25,26,27], spectrofluorimetry [28], chromatography [29,30,31,32,33,34], capillary electrophoresis [35], and voltammetry [36]. Two potentiometric methods have been reported for the determination of vildagliptin. The first sensor was a conventional liquid-contact ISE that was made of a plasticized PVC membrane cemented over a PVC tubing with an internal filling equimolar solution of vildagliptin and sodium chloride and applied hydroxy propyl β-cyclodextrin as an ionophore [37]. The other potentiometric sensor was a screen-printed carbon nanostructure thin-film Au/Pt electrode supported with a PVC membrane and used potassium tetrakis (4-chlorophenyl) borate (KTCPB) as an ion exchanger [38]. Both of them were based on the incorporation of the element of recognition into a PVC membrane that suffers some drawbacks that obviously affect the sensor’s sensitivity and stability [39].

In this study, we aimed to develop a new sensitive, accurate, and stable CPE for the precise determination of vildagliptin in bulk, pharmaceutical dosage forms, and human plasma. This proposed sensor utilized a TiO_2_–CuO bimetallic/PANI nanocomposite as a transducer in a potentiometric sensor for the first time to reach the optimum sensitivity, selectivity, accuracy, and reproducibility during measurements.

## 2. Experimental Section

### 2.1. Apparatus

The equipment used included a CLEAN digital ion analyzer PH 600, model 007747 (China); a Ag/AgCl double junction reference electrode (Thermo-Orion), model 900201; and a Heidolph MR Hei-Standard magnetic stirrer, model 100818877.

### 2.2. Chemicals and Materials

The reagents and chemicals used in our study were of analytical grade. Copper oxide CuO, titanium oxide TiO_2_, *N, N*-dimethylformamide (*N, N*-DMF), aniline monomer, and graphite powder were purchased from Sigma Aldrich, St. Louis, USA. Potassium tetrakis (4-chlorophenyl) borate (KTCPB) and 18-crown-6-ether were purchased from Acros Organics. Tetrahydrofuran (THF) was obtained from Fluka Chemical. The standard materials of vildagliptin, saxagliptin HCl, metformin HCl, ibuprofen, and diclofenac potassium were kindly supplied by the Experiments and advanced research unit, Cairo, Egypt. All the chemicals were of analytical-grade purity. Britton–Robinson buffer solutions were prepared by mixing 40 mM of phosphoric acid, acetic acid, and boric acid. The pH was adjusted using 0.2 M NaOH.

### 2.3. Standard Solutions

A standard solution of vildagliptin (1 × 10^−1^ M) was prepared by dissolving 3.03 g of standard vildagliptin in 10 mL of ethanol and completing to 100 mL volume using Britton–Robinson buffer (pH 5). The working solutions of vildagliptin were then generated by diluting the stock solution using Britton–Robinson buffer (pH 5) to obtain concentrations ranging from 1 × 10^−2^ M to 1 × 10^−10^ M.

### 2.4. Procedure

#### 2.4.1. Preparation of (TiO_2_/CuO) Bimetallic/Polyaniline Nanocomposite

The bimetallic/PANI nanocomposite was prepared using an in situ chemical oxidative polymerization method in the presence of ammonium persulphate as an oxidant in aqueous hydrochloric acid under constant stirring at 0–4 °C.

About 0.1 M of aniline hydrochloride was dissolved in 100 mL of 1 M HCl solution under continuous stirring for half an hour at room temperature. Then, a (1:1 % *w*/*w*) mixture of TiO_2_/CuO nanoparticles (0.5 g, 1 g, 2 g, and 4 g) were added to the reaction mixture and stirred thoroughly. The solution was kept at 0–4 °C in an ice bath under continuous stirring. A total of 0.1 M of ammonium persulfate solution was prepared and added drop by drop to the above solution, which was then subjected continuous stirring for 12 h to ensure complete polymerization. The solution turned blue-green and got darker as the precipitate developed. The formed product was filtered and washed several times with nearly 50 mL of HCl to remove the unreacted aniline, followed by 100 mL of water and 30 mL of acetone. The washed precipitate was dried under vacuum for 6 h at 65 °C. The Bimetallic/PANI nanocomposite, which had various TiO_2_/CuO nanoparticle to aniline monomer weight ratios (1:2, 1:1, 2:1, 4:1), was synthesized using the same procedure.

#### 2.4.2. Fabrication of the Proposed CPE

**Scheme 1:**
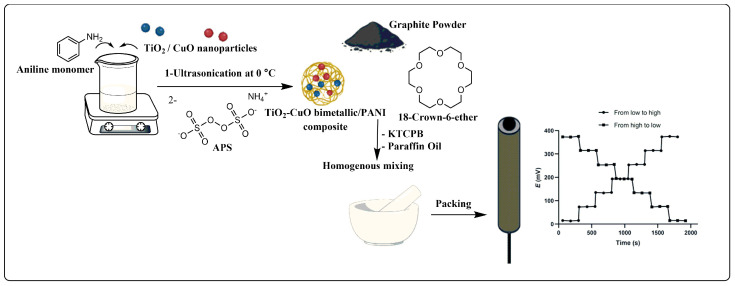
Schematic illustration of the fabrication protocol of the proposed CPE.

The working CPE was prepared by filling the prepared carbon paste into one end of a metallic spacer. The optimum electrochemical response was obtained with 50:30:10:7: 3% *w*/*w* of graphite, paraffin oil, bimetallic/PANI nanocomposite, 18-crown-6-ether, and KTCPB, respectively. The new surface of the paste electrode surface was simply conditioned in 1 × 10^−4^ M of vildagliptin aqueous solution for 1 h before starting the measurement. Scraping off about 2–3 mm of the used surface and polishing the new surface with a piece of tracing paper can regenerate the electrode surface. A schematic diagram representing the preparation of the bimetallic/PANI nanocomposite and the CPE fabrication is shown in Scheme 1.

### 2.5. Selectivity Study of the Proposed CPE

Using the matched potential method, the selectivity of the CPE was investigated in the existence of co-administered drugs and other structurally related DPP-4 inhibitors. The selectivity coefficient (*K^pot^_Vildagliptin, interferent_*) was measured by determining the activity ratio of the analyte (vildagliptin) and the interfering species that produced the same potential change under identical conditions. It was calculated according to the IUPAC guidelines using the following equation [40]:

(1) $${K^{\mathrm{pot}}}_{\left(A,\;B\right)}=\frac{∆a_{A}}{a_{B}}\;$$

In this equation, ∆*a_A_* = *a’_A_* − *a_A_*, where *K^pot^_(A, B)_* stands for the selectivity coefficient. The symbols A and B are related to vildagliptin and the interfering species, respectively. *a_A_* refers to the initial background activity of the vildagliptin reference solution, and *a’_A_* is the activity of the vildagliptin that was added to the reference solution to prompt a change in the potential value; *a_B_* is the activity of the interfering species that was added to the reference solution to produce the same potential change.

### 2.6. Quantitative Analysis of Vildagliptin in Pharmaceutical Tablets and Human Plasma

Twenty Vildagluse^®^ tablets were weighed individually to determine the average weight of one tablet. A precise amount of finely ground powered tablets equivalent to 3.03 g of vildagliptin was transferred into a 100 mL volumetric flak and dissolved in Britton–Robinson buffer (pH 5) to prepare a 1 × 10^−1^ M stock solution. Further dilutions were carried out to prepare working solutions with the following concentrations: 1 × 10^−2^ M, 1 × 10^−4^ M, 1 × 10^−6^ M, 1 × 10^−8^ M.

To determine the vildagliptin concentration in human plasma, varying concentrations of standard vildagliptin were spiked into 0.5 mL of plain plasma. Protein precipitation was achieved via the addition of 0.5 mL acetonitrile, and the solution was centrifuged at 5000 rpm for 5 min. The resulting clear supernatant was transferred into 25 mL volumetric flask and diluted to the volume with Britton–Robinson buffer (pH 5) to obtain samples with the following concentrations: 1 × 10^−2^ M, 1 × 10^−4^ M, 1 × 10^−6^ M, 1 × 10^−8^ M. The impact of the plasma matrix on the potential of the CPE was calculated by using the following equation [41]:

(2) $$MF\%=100\times (\frac{S_{m}}{S_{a}}-1)$$

where *MF* stands for matrix factor; *S_a_* and *S_m_* stand for the slope of the calibration curve in the aqueous solvent and the plasma matrix, respectively.

## 3. Results and Discussion

### 3.1. Characterization of the Prepared Bimetallic/Polyaniline Nanocomposite

**Figure 2:**
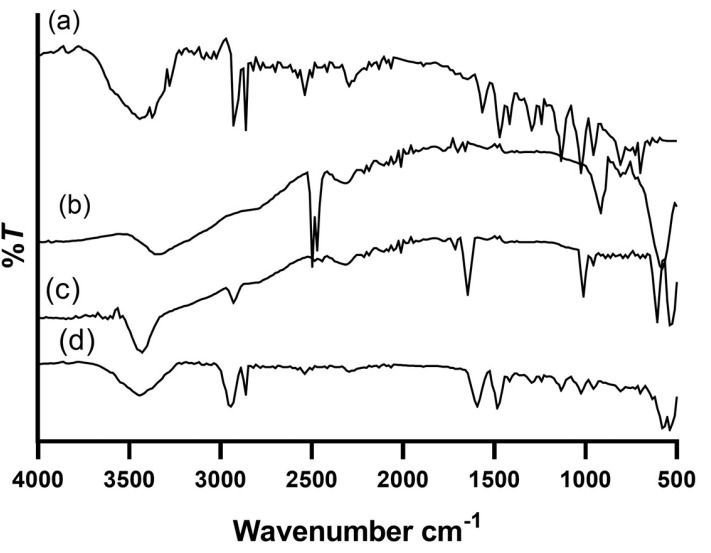
FT−IR spectra of (**a**) PANI polymer, (**b**) TiO_2_ nanoparticles, (**c**) CuO nanoparticles, and (**d**) Bimetallic/PANI nanocomposite.

Figure 2a shows the FT-IR spectrum of the polymerized aniline. This revealed absorption peaks at 1570 (strong one) and 1460 cm^−1^, which were related to the C=C stretching of quinonoid and benzenoid rings, respectively. The band at 1294 cm^−1^ is for the C-N stretching vibration of the benzenoid unit, and the band at 1024 cm^−1^ is assigned to the quinonoid unit of doped PANI. The bands at 808 and 1130 cm^−1^ are related to the aromatic C-H out-of-plane and in-plane bending vibrations, respectively. The peaks at 3430 and 2928 cm^−1^ correspond to the aromatic N-H and C-H stretching. The band at 1334 cm^−1^ is related to the C-N stretching of the primary aromatic amines. The FT-IR spectrum of TiO_2_, as shown in Figure 2b, is characterized by a broad band centred at 578 cm^−1^ due to the Ti-O-Ti stretching and bending modes. The FT-IR spectrum of the CuO nanoparticles, shown in Figure 2c, is characterized by two bands centred at 538 and 983 cm^−1^ due to the different bending vibration modes of Cu-O.

The bimetallic/PANI nanocomposite spectrum (Figure 2d) also shows the same characteristic peaks for PANI, TiO_2_, and CuO. However, compared to those of pure PANI, the corresponding peaks of the nanocomposite shifted from 1469 to 1482 cm^−1^, 1564 cm^−1^ to 1591 cm^−1^, 2928 cm^−1^ to 2941cm^−1^, and 3430 cm^−1^ to 3445 cm^−1^. These shifts may be attributed to the formation of hydrogen bonds between the TiO_2_, CuO, and NH groups of PANI on the surface of the metal oxide nanoparticles. The broad peaks positioned at 580 and 530 cm^−1^ are related to TiO_2_ and CuO, respectively. This confirms the in situ mechanism of PANI–nanocomposite formation. The intensity of most of the PANI peaks was affected by the presence of metal oxides during the polymerization of PANI, where the aniline monomers were absorbed on the metal oxide surfaces, followed by the polymerization step by the addition of ammonium persulfate. This causes the H-bonding and π–π interaction between PANI and metal oxides nanoparticles and shifts the PANI bands towards a lower wavenumber compared to pure PANI.

**Figure 3:**
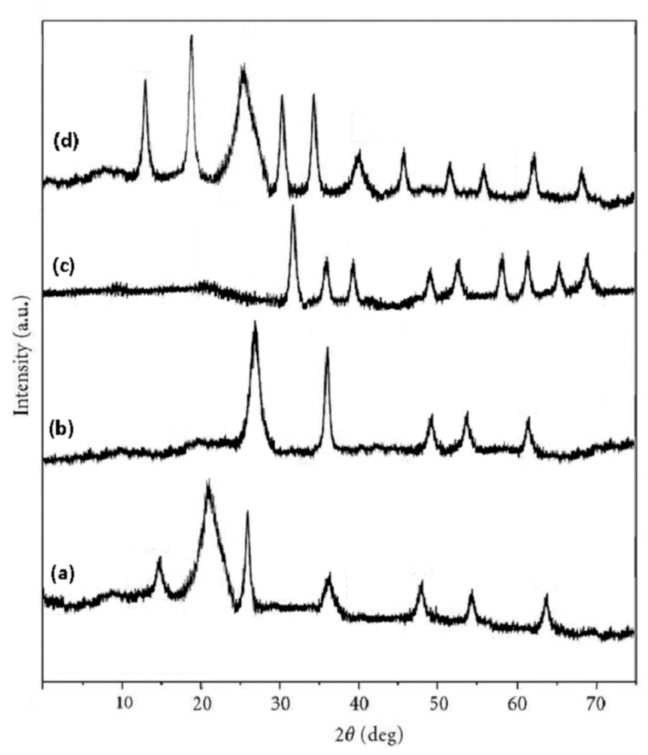
XRD pattern of (**a**) PANI polymer, (**b**) TiO_2_ nanoparticles, (**c**) CuO nanoparticles, and (**d**) Bimetallic/PANI nanocomposite.

The crystalline structure of the bimetallic/PANI nanocomposite was studied based on the XRD pattern. The XRD spectrum of PANI (Figure 3a) shows three broad peaks at 15°, 20.52°, and 25°, indicating its amorphous nature. In the case of the TiO_2_ nanoparticles (Figure 3b) the crystalline plane was indicated by the appearance of sharp peaks at 25.1°, 37.5°, 48.2°, 53.8°, and 62.2°. The CuO nanoparticles reveal peaks at 2θ 32.5°, 35.7°, 39°, 49.1°, 53.5°, 59°, 61.5°, 66.3°, and 68.2°, according to the values shown in Figure 3c. The resultant data was assigned to the crystal planes of the bodies of TiO_2_ and CuO. The XRD results are consistent with the literature [42,43,44]. Comparing the X-ray diffraction pattern of the metal oxide nanoparticles with that of the bimetallic/PANI nanocomposite (Figure 3d), the peaks become sharper with a slight shifting in position to the lower 2θ, and the degree of crystallinity increases in the case of the nanocomposite. This proves the efficient incorporation of metal oxides and their interaction with the PANI polymer matrix.

### 3.2. Potentiometric Response Features of the Studied CPEs

Reaching the optimum composition was a critical step in the fabrication of the proposed sensor. A number of CPEs with different weight percentages of their components were prepared, and their potentiometric responses were recorded (as shown in Table 1). The bare sensor was developed by homogenously mixing 70% *w*/*w* graphite with 30% *w*/*w* paraffin oil as a binder. This yielded a homogenous and robust paste. Another set of sensors were fabricated through incorporating the 18-crown-6 ionophore as a recognition element (sensors 2 and 3). This led to the observation of more linear and selective response characteristics to vildagliptin than the bare sensor. This confirmed the effective recognition of the vildagliptin molecules by the 18-crown-6 ionophore. This may be attributed to the ability of the crown ethers to form host–guest (1:1) complexes with a wide variety of cationic species in the empty cavity centre of the ring. Vildagliptin is basic in nature as it contains a secondary amino group with a pKa value of 9.03 [45] and becomes protonated at working pH. It can efficiently be recognized by the crown ether ionophore with the aid of KTCPB; a lipophilic ion exchanger facilitated the incorporation of vildagliptin into the polymeric matrix to react with the ionophore through electrostatic attraction and hydrogen bonding [22].

The effect of the transducer composition was studied via the preparation of different sensors with PANI, a TiO_2_/PANI nanocomposite, a CuO/PANI nanocomposite, and a bimetallic/PANI nanocomposite, respectively. In sensors 4, 5, and 6 different percentages of the PANI were incorporated. Generally, the addition of the transducer had a remarkable effect on the potentiometric response of the sensor. It exhibited lower response time, a better Nernstian slope, and higher sensitivity. Sensor 5, which had 10% *w*/*w* PANI, showed a better response. Sensors 7 and 8 revealed their performances after the application of 10% *w*/*w* of the TiO_2_/PANI and CuO/PANI nanocomposites, respectively. The presence of metal oxide nanoparticles in combination with PANI improved the potentiometric behaviour, the response time being minimized to 30 s. These data prove that each of the metal oxides can be applied separately in combination with PANI to act as an efficient transducer in the SC-ISEs. The preparation of the CPE with the bimetallic/PANI nanocomposite (sensor 9) showed a better response than sensors 5, 7, and 8. A synergistic effect was noticed with the presence of the two metal oxides with PANI as a transducer. They enhanced the electrode response with a wider linearity range that reached 1 × 10^−8^–1 × 10^−2^ M and a Nernstian slope of 60.04 ± 1.4 mV/concentration decade. The best response was achieved with the composition of 50:30:10:7: 3% of graphite, paraffin oil, the bimetallic/PANI nanocomposite, 18-crown-6-ether, and KTCPB, respectively.

**Figure 4:**
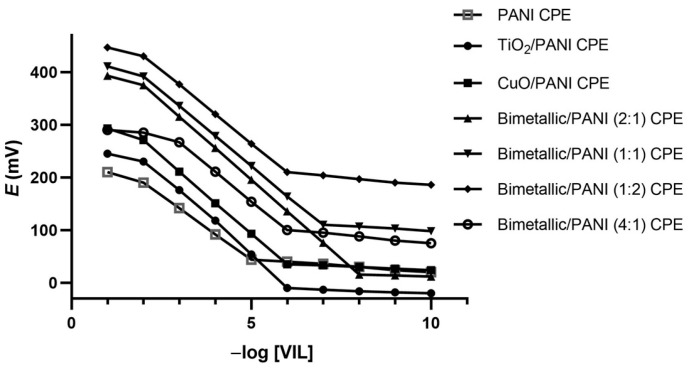
The potentiometric response of the proposed CPE using different ion-to-electron transducers.

The ratio of the bimetallic TiO_2_ and CuO to the aniline monomer may have an impact on the electrode response. This effect was studied by the formation of four sensors with different metal oxides composite/PANI ratios (1:2, 1:1, 2:1, and 4:1). As represented in Figure 4, the optimum response characteristics were observed using a nanocomposite with a bimetallic/PANI ratio of 2:1.

The CPE performance characteristics were assessed in line with the IUPAC recommendations [40], and the data are summarized in Table 2.

**Figure 5:**
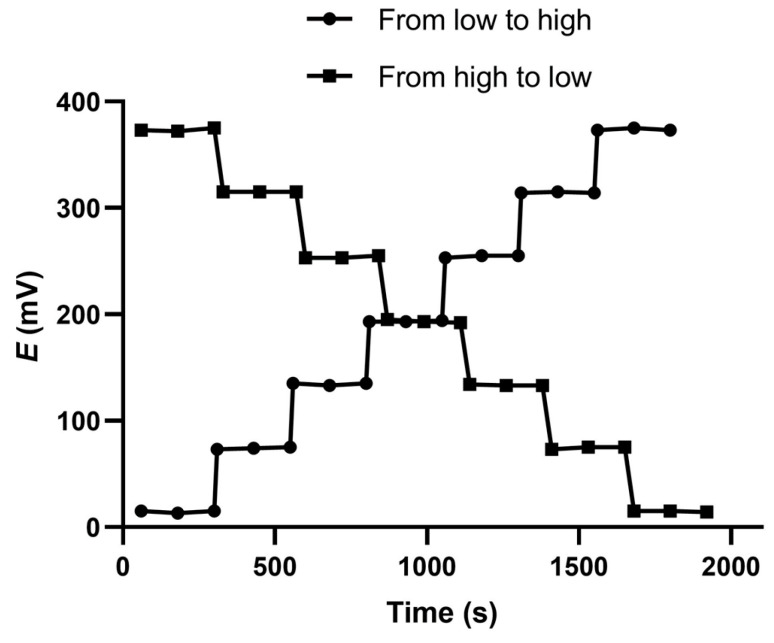
The dynamic response time of the proposed CPE (from low to high and high to low concentrations).

The proposed CPE showed superior sensitivity, as measured by the intersection of the two extrapolated linear portions of the calibration curves, which revealed a LOD value of 4.5 × 10^−9^ M. Figure 5 shows the potentiometric behaviour of the proposed sensor. It exhibits better linearity (r = 0.9997) and a faster response time (10 s ± 1.3) compared to the TiO_2_/PANI-based sensor (r = 0.9995), which had a response time of 30 s ± 5.1, and the CuO/PANI-based sensor (r = 0.9995), which had a response time (30 s ± 7.3). The faster response time of the bimetallic/PANI-based CPE is due to the induction of a more ordered transducer structure as the metal oxide nanoparticles act as fillers for the PANI polymer, leading to a large surface area that allows for higher electrical conductivity and stability.

The potentiometric response time of the CPE was investigated after a tenfold increase in vildagliptin concentration. It was found to be 10 s ± 1.3 when changing the concentration from low to high concentration values. The reversibility of the electrode response was examined upon measuring the potential of vildagliptin samples from low to high concentrations and the inverse, as shown in Figure 5. The potentiometric response was reversible and rapid, but it attained equilibrium after a longer time upon changing the concentration from high to low values (25 s ± 3).

The repeatability and inter-day precision were measured by repeatedly measuring the three vildagliptin concentrations (1 × 10^−3^ M, 1 × 10^−4^ M, 1 × 10^−5^ M) within a day and on three successive days, respectively, and the RSD% was calculated. The CPE maintained its precision, with no deviation in its potential occurring, and the RSD% was less than 1.2%. The reproducibility of the CPE was measured using three different independent fabricated sensors under the same conditions, and the potential readings of the three sensors were measured for the 1 × 10^−3^ M, 1 × 10^−4^ M, and 1 × 10^−5^ M vildagliptin solutions. The electrode shows high reproducibility (RSD% = 1.36%).

The respective stability and lifespans of the CPEs were checked by measuring their potentiometric behaviour characteristics daily to ensure that their precision was within ±2% of their original values. The variation in the calibration slope was found to be ±2.6 mV/decade after 137 days without any surface renewal.

### 3.3. The Influence of pH

**Figure 6:**
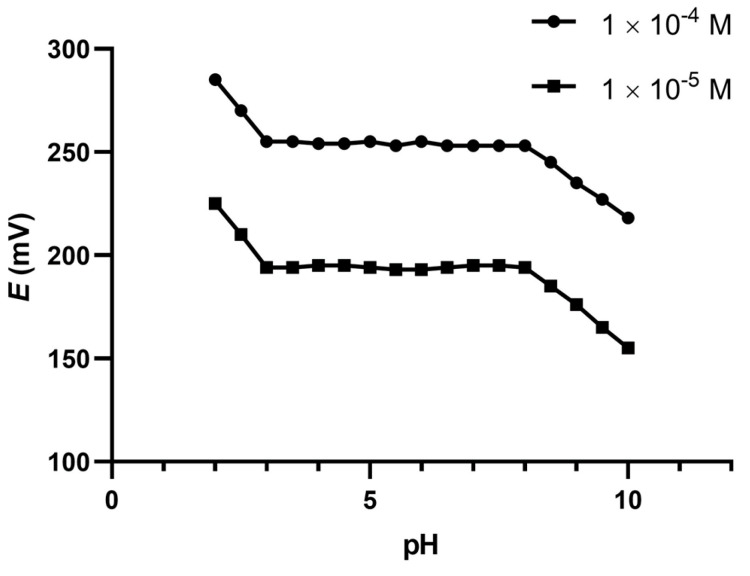
The influence of pH on the potentiometric response of the studied CPEs.

The impact of pH on the response of the proposed sensors was studied by using 1 × 10^−4^ M and 1 × 10^−5^ M vildagliptin standard solutions. The pH of the investigated solutions was adjusted in the range of 2 to 10 using aliquots of diluted hydrochloric acid or sodium hydroxide solutions. The proposed sensor showed stable, constant readings over the range 3–8, as shown in Figure 6. Therefore, pH 5 was approved as the working pH value for the proposed sensor, i.e., where vildagliptin was protonated. Above pH 8, it was observed that the potential readings decreased due to the presence of vildagliptin in a non-protonated form. Nevertheless, below pH 3, the sensors were saturated with hydrogen ions, which disturbs the performance of the sensor.

### 3.4. Selectivity Study

To evaluate the selectivity of the studied CPEs in the presence of interferents, structurally related drugs, and co-administered drugs, the matched potential method was employed. This involved adding a known amount of orphenadrine citrate solution (a_A_’) to a reference solution (1 × 10^−4^ M vildagliptin) and measuring the resulting potential change (∆E). Next, the reference solution was supplemented with a solution of an interfering ion with an activity of (a_B_) to generate an equivalent potential change (∆E), and the selectivity coefficient ($\log K_{VIL,Interferent}^{pot}$) was calculated. Table 3 lists the selectivity coefficients of the tested samples, which demonstrate the high selectivity of the CPE towards vildagliptin.

Table 4 compares the potentiometric response of the proposed CPE with that of the previously reported potentiometric sensors for vildagliptin. The results demonstrated that the suggested sensor exhibited a remarkable response to vildagliptin and demonstrated higher stability and sensitivity compared to the others. The bimetallic/PANI-based CPE showed a wider linearity range, a shorter response time, and an ideal Nernstian slope compared to the other reported sensors.

### 3.5. Water Layer Test

**Figure 7:**
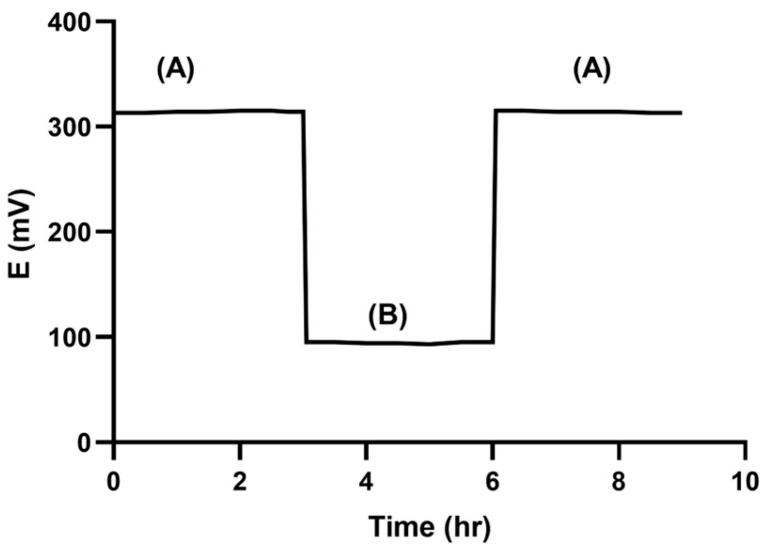
Water layer test of the proposed CPE. The potential readings at (**A**) 10^−3^ M vildagliptin, followed by (**B**) 10^−3^ M saxagliptin and (**A**) 10^−3^ M vildagliptin.

The water layer test was used to identify any possible potential drift in the response of the SC-ISEs due to the formation of a water layer between the transducer and the ISM [39]. For this test, the potential reading of 1 × 10^−3^ M of vildagliptin standard solution was monitored for 3 h as the primary ion, followed by 1 × 10^−3^ M of saxagliptin as an interfering ion for another 3 h, and then back to 1 × 10^−3^ M of vildagliptin standard solution for 3 h. Figure 7 shows that the response of the proposed sensor did not change after conditioning with the interfering ion for 3 h, indicating the absence of a water layer in the prepared sensor. This could be attributed to the hydrophobic nature of the prepared paste transducer, which prevents the formation of a water layer at the interface between the paste and the conducting surface of the CPE body.

### 3.6. Analytical Applications

The proposed sensor has been applied for the determination of vildagliptin in Vildagluse^®^ tablets and spiked samples of human plasma without any complicated pretreatment steps. As reported in Table 5, the proposed electrode exhibited high recovery values ranging from 98.87% to 100.03% and 97.65% to 99.31% for pharmaceutical tablets and human plasma, respectively. These results indicate the high efficiency and accuracy of the proposed CPE in the quantitation of vildagliptin in different matrices.

The effect of the presence of plasma endogenous components on the electrode response was studied by determining the slope of the calibration curve in the plasma matrix. It was found to be 58.87 mV/concentration decade. MF% was calculated and found to be 1.98%. This validated the absence of any interference from the plasma matrix on the CPE response.

## 4. Conclusions

In this study, a new bimetallic/PANI nanocomposite was utilized as an ion-to-electron transducer for the potentiometric determination of vildagliptin through its incorporation into carbon paste in the presence of 18-crown-6-ether as a recognition element. The electrical conductivity of the PANI was greatly enhanced by the incorporation of TiO_2_ and CuO nanoparticles. The fabricated CPE was assessed for its ability to detect vildagliptin in pharmaceutical formulations and human plasma. The sensor was studied in terms of linearity range, response time, sensitivity, and stability. It provided precise and accurate recovery values, allowing it to detect vildagliptin at concentrations as low as 1 × 10^−8^ M. The investigated sensor exhibited high selectivity and could be considered a suitable candidate for vildagliptin analysis in quality control labs.

## Figures and Tables

**Table 1:** The effect of the electrode components on the potentiometric behaviour.

	Graphite (% *w*/*w*)	PANI (% *w*/*w*)	TiO_2_/PANI Composite (% *w*/*w*)	CuO/PANI Composite (% *w*/*w*)	Bimetallic/PANI Composite (% *w*/*w*)	18-Crown-6-Ether (% *w*/*w*)	KTCPB (% *w*/*w*)	Paraffin Oil (% *w*/*w*)	Linearity Range (M)	LOD (M)	Slope (mV/Decade)	Response Time (s)
1	70	-	-	-		-		30	3 × 10^−2^–1 × 10^−1^	8 × 10^−3^	23.66	120
2	60	-	-	-	-	5	5	30	5 × 10^−3^–1 × 10^−1^	1 × 1 0^−3^	40.65	100
3	60	-	-	-	-	7	3	30	1 × 10^−3^–1 × 10^−1^	4.5 × 1 0^−4^	44.78	100
4	55	5	-	-	-	7	3	30	1 × 10^−4^–1 × 10^−2^	2 × 10^−5^	48.07	80
5	50	10	-	-	-	7	3	30	1 × 10^−5^–1 × 10^−2^	6 × 10^−6^	51.47	60
6	45	15	-	-	-	7	3	30	1 × 10^−5^–1 × 10^−3^	9 × 10^−6^	46.98	80
7	50	-	10	-	-	7	3	30	1 × 10^−6^- 1 × 10^−2^	7.5 × 10^−7^	53.91	30
8	50			10	-	7	3	30	1 × 10^−6^–1 × 10^−2^	2.2 × 10^−7^	55.26	30
9	50	-	-	-	10 (2:1)	7	3	30	1 × 10^−8^–1 × 10^−2^	4.5 × 10^−9^	60.04	10
10	50	-	-	-	10 (1:1)	7	3	30	1 × 10^−7^–1 × 10^−2^	6.5 × 10^−8^	54.88	20
11	50	-	-	-	10 (1:2)	7	3	30	1 × 10^−6^–1 × 10^−2^	8.5 × 10^−7^	52.18	40
12	50	-	-	-	10 (4:1)	7	3	30	1 × 10^−6^–1 × 10^−3^	7 × 10^−7^	55.32	25

**Table 2:** The electrochemical properties of the three CPEs with different transducers.

Parameter	PANI-Based CPE	TiO_2_/PANI-Based CPE	CuO/PANI-Based CPE	Bimetallic/PANI-Based CPE
Concentration range (M)	1 × 10^−2^–1 × 10^−5^	1 × 10^−2^–1 × 10^−6^	1 × 10^−2^–5 × 10^−6^	1 × 10^−2^–1 × 10^−8^
Slope (mV/decade)	51.47 ± 2.51	53.91 ± 1.30	55.26 ± 1.23	60.04 ± 1.14
Intercept	687.88	615.66	576.87	495.66
Correlation coefficient (r)	0.9984	0.9995	0.9995	0.9997
LOD (M)	6 × 10^−6^	7.5 × 10^−7^	2.2 × 10^−7^	4.5 × 10^−9^
Response time (s)	60	30 ± 5.1	30 ± 7.3	10 ± 1.3
Stability (days)	46	85	100	137
Working pH range	4–7	3–7	3–8	3–8
Average recovery ^1^	98.56 ± 2.03	98 ± 1.23	99.76 ± 2.87	100.76 ± 1.22
Intraday precision ^2^ (RSD%)	1.23	1.06	1.23	0.95
Interday precision ^2^ (RSD%)	1.53	1.12	1.44	1.07
Reproducibility ^3^ (RSD%)	1.72	1.45	1.68	1.36

^1^ The mean of five measurements taken at five different concentration levels. ^2^ The mean of five determinations of three QC samples. ^3^ The mean of five determinations of three QC samples using three independently fabricated sensors.

**Table 3:** Potentiometric selectivity coefficient ($\log K_{vildagliptin,\;Interferent}^{pot}$) for the proposed CPE.

Interfering Species	$\left(\log K_{Vildagliptin,\;Interferent}^{pot}\right)$
NaCl	−4.21
MgCl_2_	−4.06
Talc	−3.77
Starch	−3.52
Saxagliptin	−3.58
Alogliptin	−3.33
Metformin HCl	−4.21
Glucose	−3.65
Lactose	−3.05

**Table 4:** Comparison between the proposed CPE and other potentiometric sensors designed for detecting vildagliptin that have been documented in the literature.

Electrode Composition	Linearity Range (M)	LOD (M)	Slope (mV/Decade)	Response Time (s)	Lifetime	pH Range	Ref
Conventional LC-ISE with a plasticized PVC membrane that is formed of tetrakis p-chloro tetra phenyl borate as an ion exchanger in NPOE as a plasticizer and (2-hydroxypropyl)-β cyclodextrin as an ionophore.	1 × 10^−1^–1 × 10^−5^	5 × 10^−6^	55.3	15 s	15 days	4–8	[34]
Screen-printed carbon, nanostructure thin-film Au/Pt and Pt/Pt with a plasticized PVC membrane containing potassium tetrakis(4-chlorophenyl) borate.	1 × 10^−2^–1 × 10^−5^	2.2 × 10^−6^	64.4 ± 1	≤20 s	-	2.5–5.8	[35]
CPE with 50:30:10:7: 3% *w*/*w* of graphite, paraffin oil, bimetallic/PANI, 18-crown-6-ether, and KTCPB, respectively.	1 × 10^−2^–1 × 10^−8^	4.5 × 10^−9^	60.04 ± 1.14	10 s	137 days	4–9	This work

**Table 5:** Determination of vildagliptin in pharmaceutical formulation and spiked human plasma by the proposed sensor.

	Vildagluse^®^ Tablets		Human Plasma	
Concentration (M)	Recovery%	RSD	Recovery%	RSD
1 × 10^−3^	98.87	0.75	99.31	0.98
1 × 10^−4^	99.23	1.03	98.14	1.32
1 × 10^−5^	100.03	0.88	98.74	1.27
1 × 10^−6^	99.41	0.95	97.65	1.41
Mean ± SD	99.39 ± 0.49		98.46 ± 0.72	

## Data Availability

The data presented in this study are available on request from the corresponding author.
